# Evaluation of a Rhenium(I) Complex and Its Pyridostatin-Containing Chelator as Radiosensitizers for Chemoradiotherapy

**DOI:** 10.3390/molecules30153240

**Published:** 2025-08-01

**Authors:** António Paulo, Sofia Cardoso, Edgar Mendes, Elisa Palma, Paula Raposinho, Ana Belchior

**Affiliations:** 1 Centro de Ciências e Tecnologias Nucleares, Instituto Superior Técnico, Universidade de Lisboa, Campus Tecnológico e Nuclear, Estrada Nacional 10, Km 139.7, 2695-066 Bobadela LRS, Portugal; apaulo@ctn.tecnico.ulisboa.pt (A.P.); sofia.cardoso@tecnico.ulisboa.pt (S.C.); edgar.mendes@tecnico.ulisboa.pt (E.M.); elisa@ctn.tecnico.ulisboa.pt (E.P.); paular@ctn.tecnico.ulisboa.pt (P.R.); 2 Departamento de Engenharia e Ciências Nucleares, Instituto Superior Técnico, Universidade de Lisboa, Estrada Nacional 10, Km 139.7, 2695-066 Bobadela LRS, Portugal; 3 Departamento de Física, Instituto Superior Técnico, Universidade de Lisboa, Av. Rovisco Pais, 1049-001 Lisboa, Portugal

**Keywords:** Re(I) complexes, pyridostatin derivatives, G4-DNA binders, radiosensitizers, Co-60 irradiation

## Abstract

The use of radiosensitizers is a beneficial approach in cancer radiotherapy treatment. However, the enhancement of radiation effects on cancer cells by radiosensitizers involves several different mechanisms, reflecting the chemical nature of the radiosensitizer. G-quadruplex (G4) DNA ligands have emerged in recent years as a potential new class of radiosensitizers binding to specific DNA sequences. Recently, we have shown that the Re(I) tricarbonyl complex PDF-Pz-Re and its pyrazolyl-diamine chelator PDF-Pz, carrying a *N*-methylated pyridostatin (PDF) derivative, act as G4 binders of various G4-forming DNA and RNA sequences. As described in this contribution, these features prompted us to evaluate PDF-Pz-Re and PDF-Pz as radiosensitizers of prostate cancer PC3 cells submitted to concomitant treatment with Co-60 radiation. The compound RHPS4 was also tested, as this G4 ligand was previously shown to exhibit strong radiosensitizing properties in other cancer cell lines. The assessment of the resulting radiobiological effects, namely through clonogenic cell survival, DNA damage, and ROS production assays, showed that PDF-Pz-Re and PDF-Pz were able to radiosensitize PC3 cells despite being less active than RHPS4. Our results corroborate that G4 DNA ligands are a class of compounds with potential interest as radiosensitizers, deserving further studies to optimize their radiosensitization activity and elucidate the mechanisms of action.

## 1. Introduction

External beam radiation therapy (EBRT) is an essential cancer treatment, which makes use of different types of ionizing radiation (IR), such as ɣ- or X-rays, high-energy electrons or hadrons (e.g., protons and carbon ions). In the past few years, EBRT underwent important technological developments, namely with the introduction of the intensity modulated radiation therapy and image-guided radiation therapy modalities, which allowed more accurate and precise radiation dose deposition in the target tumor tissues and fostered more efficient treatments [1,2]. Despite this progress, the undue irradiation of nearby healthy tissues with harmful doses of radiation still is a limitation in many EBRT treatments. Tumor radioresistance is another issue inherent to radiation therapies that strongly limits their therapeutic success, particularly for photon beam radiotherapy [3]. To overcome these drawbacks, the use of radiosensitizers in combination with radiation therapy has emerged as a promising strategy. This approach has been thoroughly investigated using a wide variety of molecules, materials, and radiation types, including both preclinical studies and some clinical trials [4,5,6]. The radiosensitizer can be considered a drug that enhances the tumor cell killing ability in a synergistic way when combined with IR, i.e., leading to a biological response greater than that expected by the additive effect of IR exposure and treatment with the chemical drug.

Radiosensitizers enhance the effects of radiation on cancer cells through diverse mechanisms, shaped by their chemical properties, specific biological pathways, and targets. DNA-damaging agents correspond to an important class of radiosensitizers and prominent examples include the nucleoside analogue gemcitabine and the DNA-alkylating and platinating agents temozolomide and cisplatin, respectively, which are in clinical use as anticancer drugs [7,8,9]. Alternatively, compounds that are able to inhibit DNA damage repair pathways are also potentially useful radiosensitizers that increase the number of unrepaired IR-induced DNA lesions with consequent enhancement of radiotoxicity. A relevant example is the poly (ADP-ribose) polymerase (PARP) inhibitor Olaparib, which is an approved targeted cancer drug undergoing current clinical trials as a radiosensitizing agent [10]. A variety of other classes of molecules interfering with the cell cycle regulation and normoxic/hypoxic status of cancer cells have also been studied as radiosensitizers. In addition to the previously mentioned classes of radiosensitizers involved in biological processes that enhance the radiosensitivity of cancer cells, high atomic number (Z) materials—such as gold nanoparticles or iodinated contrast agents—can also function as radiosensitizers through physical mechanisms that locally increase the radiation dose within cancer cells pre-treated with these compounds [4,11]. Such effects result from the emission of secondary electrons by the high-Z materials inside the cancer cells, through internal conversion or Auger processes, upon their exposure to IR.

Among DNA-binding compounds, G-quadruplex (G4) ligands have more recently emerged as a potential new class of radiosensitizers, targeting specific DNA sequences. G4 structures are formed by guanine-rich DNA or RNA sequences through the stacking of at least two G-tetrads—square planar arrangements of four guanines stabilized by Hoogsteen hydrogen bonds and monovalent cations such as potassium (K^+^) and sodium (Na^+^) [12]. G4 structures are particularly prevalent in the telomeres and the promoter regions of oncogenes, within critical regions of the DNA or RNA that influence gene metabolism processes and key biological processes such as DNA replication and recombination, gene expression, and telomere protection. Consequently, a wide variety of G4 ligands, acting namely as telomerase inhibitors or oncogene repressors, have been investigated as potential anticancer drugs with encouraging biological results. Many of these ligands have been shown to induce DNA damage, promote cell cycle arrest, interfere with DNA damage response (DDR) and, ultimately, exert antiproliferative effects on target cancer cells [12].

**Figure 1:**
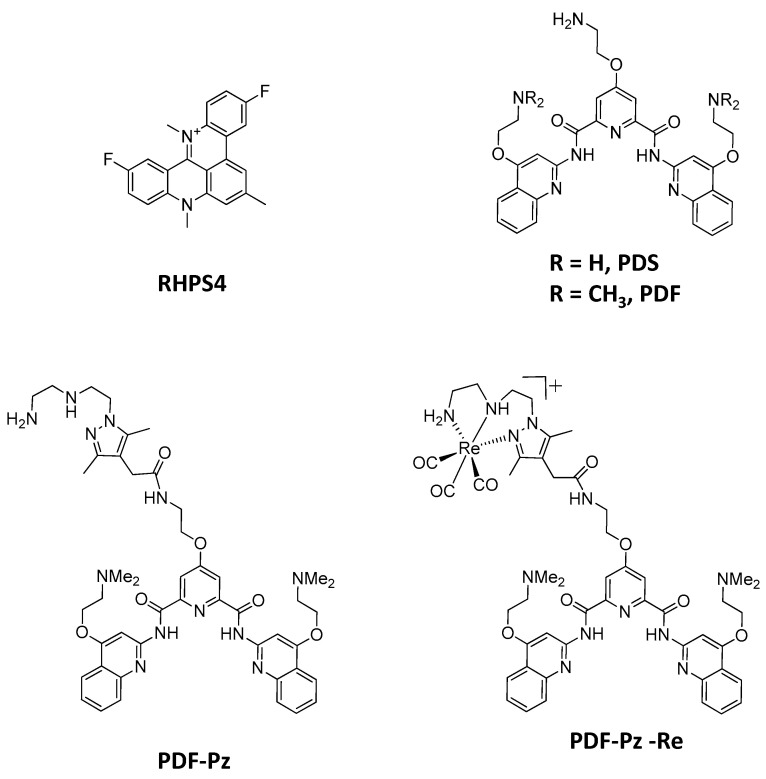
Structures of the G4-DNA ligands evaluated in this work as radiosensitizers of PC3 PCa cells.

The first study investigating the radiosensitizing activity of G4 ligands in cancer cells was reported by Merle et al. and involved *N*-methylated derivatives of 4,6-bis-(6-(acrid-9-yl)-pyridin-2-yl)-pyrimidine [13]. The study showed that the combined treatment of glioblastoma (GBM) cells with nontoxic concentrations of the compounds and 2 Gy X-ray irradiation led to the radiosensitization of the cells in a concentration-dependent manner. Later on, the same authors evaluated the radiosensitizing properties of a chlorido Pt (II) complex containing a tridentate terpyridine derivative (Pt-ctpy) [14]. Pt-ctpy was able to radiosensitize GBM and non-small-cell lung cancer cell lines following X-ray irradiation, and it also significantly reduced tumor growth in nude mice with GBM xenografts. RHPS4 (3,11-difluoro-6,8,13-trimethyl-8H-quino [4,3,2-kl] acridinium methosulfate) (Figure 1), one potent G4-DNA ligand, acts also as an efficient radiosensitizer of GBM cells, upon exposure to photon irradiation or to carbon ion beams, according to in vitro studies [15,16,17,18]. Furthermore, RHPS4 demonstrated to be a potent radiosensitizer in vivo in a GBM xenograft mouse model, within preclinical radiotherapeutic assays comprising the combined treatment with RHPS4 and X-rays [19]. Overall, these findings highlight the potential of combining G4 ligands with IR in cancer chemoradiotherapy, although studies in this area remain scarce [20]. The radiosensitizing activity of the tested G4 ligands seems to be related to telomere disfunction, disruption of cell cycle progression, and interference with DDR pathways—mechanisms that are strongly influenced by the chemical nature of each ligand.

In this context, we decided to proceed with the evaluation of the radiosensitizing ability of the Re(I) complex PDF-Pz-Re and its tridentate chelator PDF-Pz (Figure 1) in prostate cancer (PCa) PC3 cells submitted to post-irradiation with Co-60 gamma photons. PDF-Pz-Re carries a *N*-methylated pyridostatin derivative and we have shown previously that it can stabilize G4-structures from different DNA or RNA sequences, such as those present on the SRC proto-oncogene and telomeric RNA [21]. Interestingly, the possibility of preparing the congener ^99m^Tc complex, PDF-Pz-^99m^Tc, allows theranostic chemoradiotherapy approaches based on PDF-Pz-Re, as ^99m^Tc is the most used radionuclide for imaging by Single-Photon Emission Computed Tomography (SPECT) in nuclear medicine [21,22]. We have shown that PDF-Pz-^99m^Tc can be internalized by prostate cancer (PCa) PC3 cells while PDF-Pz-Re exhibited a moderate cytotoxicity in the same cell line. These results justified the selection of PC3 cells for the first evaluation of PDF-Pz-Re as a potential radiosensitizer. Furthermore, EBRT has well-recognized importance in PCa treatment and PCa is prone to radioresistance that can be mitigated by the use of radiosensitizers [23,24].

In this study, we describe irradiation experiments on PC3 PCa cells using Co-60 ɣ photons, including both IR-only protocols and combined treatments with IR and either PDF-Pz-Re or its chelator, PDF-Pz. Post-irradiation cell survival assays were conducted to evaluate the radiosensitizing potential of the tested compounds. For comparative purposes, the experiments were extended to include the parental pyridostatin derivatives (PDS and PDF), as well as RHPS4 that remains one of the most potent G4-DNA binding radiosensitizers. To gain deeper insights into the radiosensitizing mechanisms of PDF-Pz-Re and PDF-Pz, additional assays were performed to assess DNA damage and reactive oxygen species (ROS) production in PC3 cells treated with the compounds and/or Co-60 ɣ photon irradiation.

## 2. Results and Discussion

### 2.1. Cytotoxicity Studies: Selection of the Concentration of Radiosensitizers

**Figure 2:**
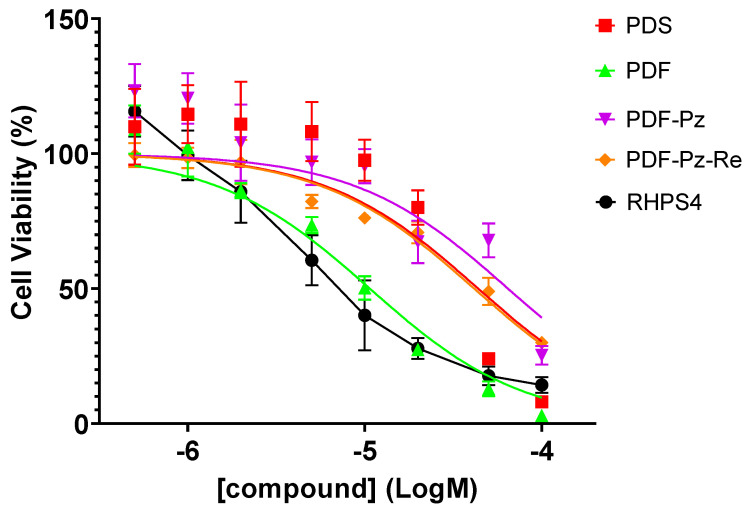
Cellular viability of PC3 cells after 48 h of incubation with the compounds PDS, PDF, PDF-Pz, and PDF-Pz-Re [21] and with RHPS4 at different concentrations (0.5–100 µM). Data are expressed as the percentage of cellular viability (mean ± SEM).

In a chemoradiotherapy approach, it is important to use a sub-cytotoxic concentration of the chemical drug to take advantage of the combined effect with the IR treatment and minimize undesired toxic effects [4]. As mentioned above, we have previously reported that PDF-Pz-Re and its chelator PDF-Pz exhibited a moderate cytotoxic activity in PC3 cells, similarly to the parental compounds PDS and PDF (Figure 2) [21]. The cytotoxic activity of PDF-Pz-Re in this cell line is very similar to that we have previously found for the classical anticancer drug cisplatin under the same conditions with IC50 values of 41.6 ± 8.8 and 48.8 ± 1.2 μM, respectively [25].

In this work, we also evaluated the cytotoxic effect of RHPS4 in PC3 prostate cancer cells using the MTT (3-(4,5-dimethylthiazol-2-yl)-2,5-diphenyltetrazolium bromide) assay, under the same conditions previously applied to the different pyridostatin derivatives, i.e., after 48 h of incubation at 37  °C. RHPS4 exhibited a cytotoxic effect in PC3 cells (IC50 = 8.7 ± 1.9 µM) comparable to that of PDF (IC50 = 10.7 ± 2.2 µM), which is the most active pyridostatin derivative in this cell line.

Based on these data, and in order to avoid chemical toxicity effects, we selected concentrations of each compound below their IC50 values, ranging from 5 to 30 µM (see Table 1), to perform the radiosensitization studies described below.

### 2.2. Assessment of Radiosensitization Effects by the Clonogenic Survival Assay

Radiosensitizing effects were evaluated using the clonogenic survival assay, which accurately reflects the inhibition of cell proliferation following exposure to IR. For this purpose, cells were exposed to pre-established doses of radiation, either in the presence or absence of the candidate radiosensitizer, applied at sub-cytotoxic concentrations as previously mentioned. The effects of PDS, PDF, PDF-Pz, PDF-Pz-Re, and RHPS4 on the proliferation of PC3 cells in combination with ɣ-radiation were assessed for doses ranging from 0 to 6 Gy. The rationale for selecting these doses is based on clinical radiotherapy practices, where conventional fractionation typically involves doses ranging from 1.8 to 2 Gy per fraction, while hypofractionation protocols use higher doses, generally between 2.5 and 5 Gy per fraction [26].

**Figure 3:**
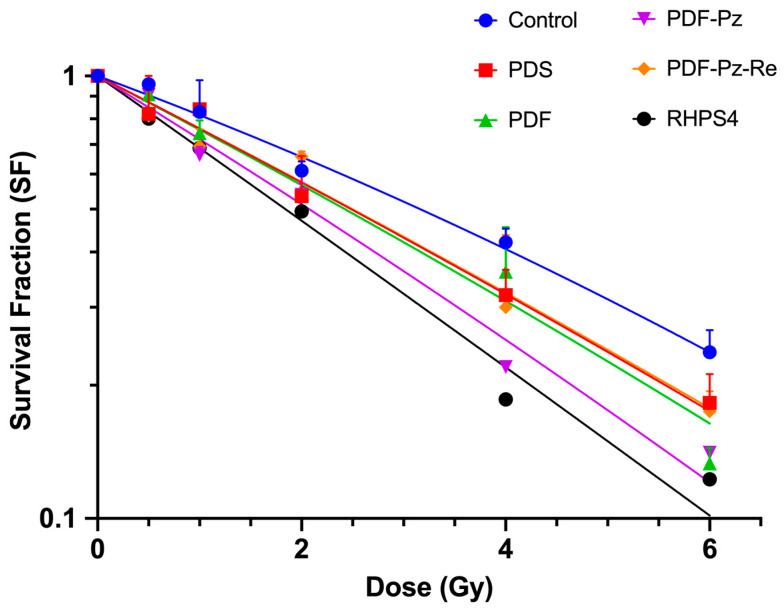
Inhibitory effect of PDS (20 µM), PDF (5 µM), PDF-Pz (30 µM), PDF-Pz-Re (30 µM), and RHPS4 (2.5 µM), in combination with Co-60 irradiation, on the proliferation of PC3 cells. After incubation with the compounds for 48 h, PC3 cells were exposed to 0.5, 1, 2, 4, and 6 Gy of γ-radiation (dose rate of 1 Gy/min). IR-only control experiments were also performed for each tested radiation dose. The results were calculated from independent biological replicates (n = 3) and are given as the mean ± SEM.

The results are presented in Figure 3, alongside those from the corresponding control experiments (without added radiosensitizer) in the same range of radiation doses.

In all experiments, the survival fraction (SF) decreased progressively with increasing radiation doses. However, the SF curves obtained in the presence of the tested compounds demonstrated a greater inhibition of cell proliferation compared to the IR-only control, suggesting that all compounds exerted a radiosensitizing effect on PC3 cells. The experimental survival data were fitted using the linear-quadratic (LQ) model, according to eqn. (1) (see Section 3) [27]. The best-fit curves (Figure 3) were used to determine the model parameters α [Gy^−1^] and β [Gy^−2^], which are listed in Table 2.

The LQ parameters α and β are related with cellular radiosensitivity and describe how radiation affects cells across different dose levels. The α parameter is related to the probability of a lethal event occurring through a single-track mechanism, and β with the probability of sublethal damage occurring resulting from the interaction of two independent radiation tracks. Generally, the linear α term predominates at low doses, whereas the quadratic β term becomes more relevant at higher doses. The α and β values determined for PC3 cells under IR-only conditions (see Table 2) are consistent with those previously described by other authors for this cell line, upon X-ray irradiation, and are in line with its known intrinsic radioresistance [28].

Treatment of the cells with the G4 ligands resulted in an increase of the α parameter, appearing in the range 0.270–0.375 Gy^−1^, when compared to the control IR-only experiment (α = 0.197 Gy^−1^). This trend confirmed the radiosensitizing ability of the compounds. In contrast, the β parameter in the combined treatment regimen ranged from 0.001 to 0.005 Gy^−2^, slightly lower than that observed in the control experiment (β = 0.007 Gy^−2^). The low β values in the presence of the G4 ligands, along with the associated large uncertainties, indicates that the variation in the survival fraction as a function of dose is better adjusted by a linear model rather than a quadratic one. This behavior is a signature of high-LET radiation and is consistent with the radiosensitization of the cells by the different compounds, although to a different extent.

To have a clearer insight into the radiosensitization capabilities of the evaluated G4 ligands, the D_10_ values were estimated using the calculated α and β parameters. D_10_ is a radiosensitivity parameter that corresponds to the radiation dose leading to 10% cell survival fraction [29]. In the absence of G4 ligands, i.e., for PC3 cells submitted to the IR treatment alone, a D_10_ value of 8.9 Gy was obtained. In contrast, lower D_10_ values were calculated in the presence of the G4 ligands, ranging from 6.1 to 7.8 Gy (Table 2). These results indicate that irradiation in the presence of the compounds more efficiently reduces cell survival, thereby confirming their radiosensitizing properties.

The compound RHPS4 exhibited superior radiosensitizing properties compared to the pyridostatin derivatives, as evidenced by its highest enhancement factor at the D_10_ dose (EF(D_10_)): 1.5 for RHPS4 versus values ranging from 1.1 to 1.4 for the pyridostatin derivatives (Table 2). The parental PDF compound showed an EF(D_10_) value of 1.2 that increased to 1.4 in the case of the pyrazolyl-diamine chelator PDF-Pz and decreased to 1.1 for the respective Re(I) complex PDF-Pz-Re. The enhancement factor obtained for parameter α (EF(α)) ranges from 1.4 to 1.9, whereas the enhancement factor for β (EF(β)) varies between 0.1 and 0.4. This reveals an increase in DNA damage by single tracks, which is a high-LET radiation signature. The complex PDF-Pz-Re showed a reduced EF compared to PDF-Pz.

The comparison of the radiosensiting capability of PDF-Pz-Re with other d-transition metal complexes is not a straightforward task, as the reported studies are scarce and involve different cell lines and different irradiation conditions. Recently, it has been described that Re(I) tricarbonyl complexes carrying a quinazoline derivative are able to radiosensitize A431 carcinoma cells to Co-60 radiation [30]. However, the authors did not quantify the corresponding EF values. As mentioned above, the Pt(II) complex Pt-ctpy, acting also as a G4-binder, was shown to radiosensitize human glioblastoma (SF763 and SF767) and non-small-cell lung cancer (A549 and H1299) cells with dose-enhancement factors ranging between 1.32 and 1.77 [14], which are higher than the EF value of 1.1 obtained by us for PDF-Pz-Re. However, PDF-Pz-Re still demonstrated promising radiosensitizing activity, which warranted the study of additional radiobiological endpoints—such as DNA damage and ROS production—as detailed below.

Several factors can justify the trend observed for the radiosensitizing ability of the different G4 ligands tested. First of all, the degree of cellular uptake of the compounds is a crucial factor influencing the extent of the biological processes underlying radiosensitization of the PC3 cells. Using the congener ^99m^Tc-labeled complex of PDF-Pz-Re, we confirmed that this class of complexes can be uptaken by PC3 cells, based on quantitative gamma-counting measurements [21]. For the other G4 ligands, the determination of their cellular uptake is not straightforward being organic compounds and lacking a readily traceable radiolabel. Nevertheless, it is reasonable to consider that they might have a higher cellular uptake than PDF-Pz-Re, showing less favorable higher molecular weight and topology to diffuse through the cell membrane and, thus, eventually accumulating less in the cells and acting as a less efficient radiosensitizer.

As mentioned above, the radiosensitization activity of G4 ligands seems to be related to the induction of telomere disfunction and interference with cell cycle progression, among other biological effects. It has been reported that RHPS4 tends to arrest cancer cells at the G2/M phase [15]. By contrast, we have verified that the pyridostatin derivatives under study lead to cell cycle arrest of PC3 cells in phase G0/G1 [21]. It is well recognized that different phases of the cell cycle display different cellular radiosensitivity. G2 and mitotic (M) phases are considered the most sensitive to radiation followed by G1, while S phase cells are comparatively resistant [31]. Altogether, these biological features corroborate the radiosensitization effect observed for all tested G4 ligands, and likely justify that RSHP4 acted as the most effective radiosensitizer of PC3 cells exposed to Co-60 gamma radiation.

### 2.3. DNA Damage: ɣ-H2AX Assay

**Figure 4:**
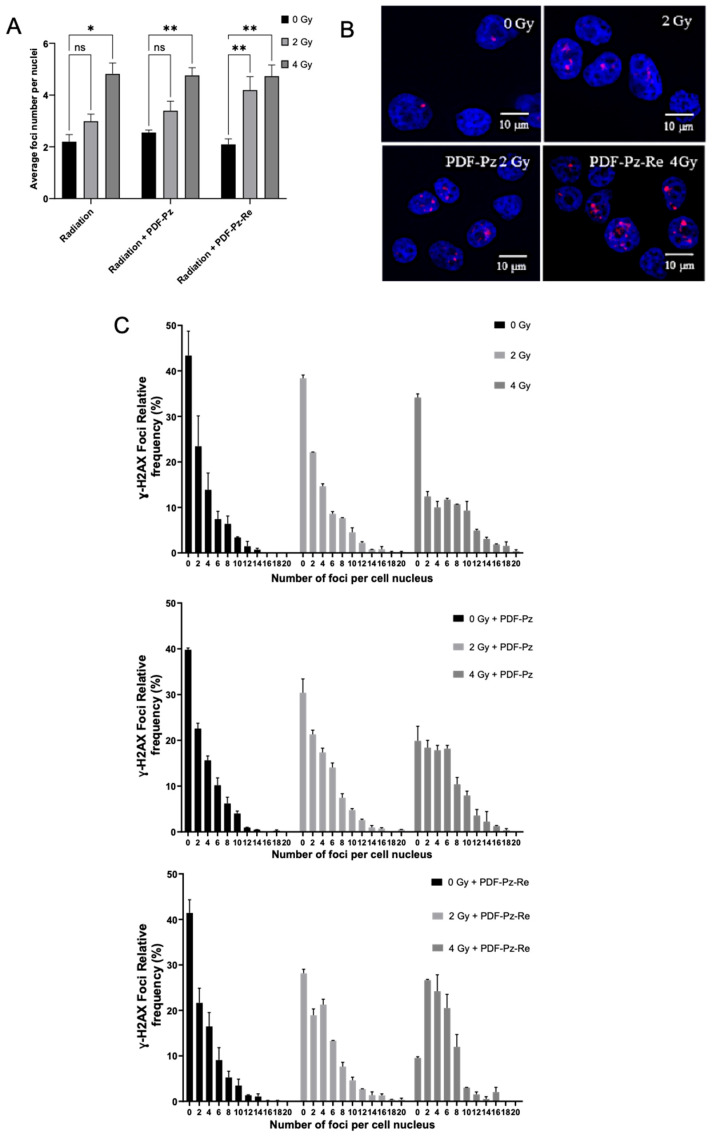
DNA damage induced in PC3 cells treated with Co-60 irradiation at 2 and 4 Gy doses after previous incubation with 30 μM of PDS-Pz and PDS-Pz-Re, for 48 h at 37 °C. Control cells were incubated with medium only. (**A**) Quantification of γ-H2AX foci per nucleus (mean ± SEM, 2 independent experiments), expressed as the average number of foci. Statistical differences compared to the control are denoted as not significant (ns) and * *p* < 0.05, ** *p* < 0.01 (**B**) Representative fluorescence images of cells exposed to the different radiation doses in the presence or not of the tested compounds. (**C**) Distribution of γ-H2AX foci across samples, presented as the relative frequency of nuclei with a certain number of foci.

To gain further insight into the radiosensitizing properties of PDF-Pz-Re and its chelator PDF-Pz, we have studied their influence on the genotoxic effects in PC3 cells exposed to Co-60 irradiation. The study was done through the ɣ-H2AX assay that quantifies γ-H2AX foci, a well-established marker for DNA double-strand breaks (DSBs), an important radiobiological endpoint [32]. For this study, we have considered 2 and 4 Gy doses and sub-cytotoxic concentrations of the compounds, as in the clonogenic survival assays. DNA damage assessment was performed immediately after the irradiation of the cells with Co-60 gamma rays, and the results are presented in Figure 4.

As shown in Figure 4A, the non-irradiated cells, i.e., for 0 Gy, showed a similarly low average number of foci, either when treated or not with PDF-Pz or PDF-Pz-Re. This result confirmed that the compounds are unable to induce significant DNA damage at the sub-cytotoxic concentrations used in the assays. The exposure of PC3 cells to 2 and 4 Gy, without previous incubation with the compounds, led to an increase in the average foci number per nuclei when compared with its own control, being statistically significant (* *p* < 0.05) at 4 Gy. The irradiation of the cells previously treated with 30 µM of PDF-Pz or PDF-Pz-Re led to a modest increase in the average foci number, rather similarly to the corresponding IR-only experiments (Figure 4A). For the 2 Gy dose experiments, however, a slightly more intense increase in the foci number was observed for the cells treated with PDF-Pz and PDF-Pz-Re when compared with the cells treated uniquely with the same IR dose, as shown for PDF-Pz in Figure 4B. At this same dose, PC3 cells previously treated with the G4 ligands present fewer cells with zero foci than the ones submitted only to Co-60 irradiation (Figure 4C), although this increase is not statistically significant. This result seems to indicate that PDF-Pz and PDF-Pz-Re were able to enhance DNA damage upon irradiation of the cells. In the case of the 4 Gy dose, the DNA damage in the cells treated only with IR irradiated and in those treated also with the compounds was essentially the same. However, for this higher dose, the presence of the compounds has also contributed to a decrease in the number of cells with zero foci when compared with the counterpart IR-only experiments. Our results are consistent with previous reports for different G4 ligands in other cancer cell lines, which usually led to a modest enhancement of radiation-induced DNA damage [15].

### 2.4. ROS Production

In addition to the DNA damage induced by direct effects of IR, DNA can also be damaged indirectly through the generation of reactive oxygen species (ROS) that result from the interaction of radiation with water, the most abundant molecule in cells. The interaction of IR with water generates free radicals such as hydroxyl (OH⦁), hydrogen (H⦁), and the hydrated electron (e^−^ (aq)). Among these, OH⦁ is particularly reactive and can interact with DNA bases and the sugar–phosphate backbone through various mechanisms, leading to a wide range of modifications collectively referred to as oxidative DNA damage. Other ROS species, such as superoxide anions (O2•−) and hydrogen peroxide (H_2_O_2_), may also contribute to this damage. Notably, the indirect effects of radiation are estimated to cause approximately three times more DNA damage than direct effects [33]. Thus, the assessment of ROS production can give valuable hints to better interpret radiobiological effects.

**Figure 5:**
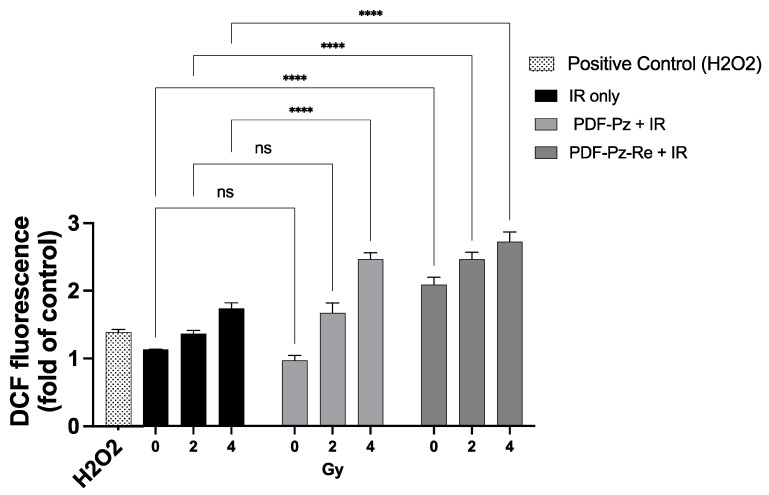
ROS production in PC3 cells treated with PDF-Pz or PDF-Pz-Re (30 µM). Cells were incubated with the compounds and then irradiated at 2 and 4 Gy. Similar experiments were run for cells treated uniquely with IR, at the same radiation doses. Cells treated with H_2_O_2_ (10 μM) were used as a positive control. ROS production was measured 1 h post-irradiation. Non-irradiated cells (0 Gy) under IR-only conditions served as the control group (not significant (ns); **** *p* ≤ 0.0001). The results were calculated from independent biological replicates (n = 3) and are given as the mean ± SEM.

As discussed above, the compounds PDF-Pz and PDF-Pz-Re slightly to moderately increased the DNA damage in PC3 cells upon their irradiation with Co-60 photons. Thus, we have investigated whether this increase could be correlated with the influence of the compounds on the production of ROS. For this study, PC3 cells were incubated with PDF-Pz and PDF-Pz-Re at sub-cytotoxic concentrations (30 µM), treated with 2 and 4 Gy of Co-60 irradiation and thereafter the production of ROS was quantified by a fluorescent assay using 2′,7′-dichlorodihydrofluorescein diacetate (H2DCF-DA) as a fluorogenic probe, which is commonly used to determine total cellular ROS [34]. The corresponding IR-only experiments were also performed. The results are presented in Figure 5.

After irradiation, the results showed an increase in the number of ROS with increasing dose values, for the all conditions tested (Figure 5). At 4 Gy, the number of ROS produced after incubation with PDF-Pz and PDF-Pz-Re were significantly higher when compared with those produced in the respective IR-only experiment (**** *p* ≤ 0.0001). At 2 Gy, however, only the PDF-Pz-Re compound was able to enhance the production of ROS after cell irradiation (**** *p* ≤ 0.0001).

Remarkably, unlike PDF-Pz, the compound PDF-Pz-Re seemed to contribute to the production of ROS without irradiation of the cells, as an increase of intracellular ROS levels was detected when compared with control cells not submitted to the treatment with radiation or with the compound (**** *p* < 0.0001). This finding indicates that the presence of the Re(I) tricarbonyl core might influence the redox status of the PC3 cells with consequent elevation of the ROS levels. A similar behavior was reported by other authors for other organometallic Re(I) tricarbonyl complexes in different tumor cells [35,36]. Despite this result, PDF-Pz-Re induced lower radiosensitizing effects than its chelator (PDF-Pz). Thus, other factors should play an important role in the radiosensitization activity of the compounds, eventually related with their G4-DNA binding properties that can influence cell cycle progression and DDR processes. It is important to mention that there is increasing evidence that G4 structures are present in mitochondrial DNA and some G4-binders were shown to interfere with mitochondrial function [37], which might play a role on the radiosensitization activity of this class of compounds.

## 3. Materials and Methods

### 3.1. G4 Ligands

The compounds 4-(2-aminoethoxy)-N2,N6-bis [4-(2-aminoethoxy)-2-quinolinyl]-2,6-pyridinedicarboxamide hydrochloride (PDS) (purity: ≥98%; C_31_H_32_N_8_O_5_ xHCl; MW = 596.64 gmol^−1^ (free base)), and 3,11-difluoro-6,8,13-trimethyl-8H-quino [4,3,2-kl] acridinium methosulfate (RHPS4) (purity: ≥98%; C_22_H_17_F_2_N_2_·CH_3_O_4_S; MW = 458.48 gmol^−1^) were purchased from Sigma-Aldrich (St. Louis, MO, USA) and used without further purification. The pyridostatin derivatives 4-(2-aminoethoxy)-N2,N6-bis(4-(2-(dimethylamino)ethoxy)quinolin-2-yl)pyridine-2,6-dicarboxamide (PDF) (C_35_H_40_N_8_O_5_; MW = 652.74 gmol^−1^), 4-(2-(2-(1-(2-((2-aminoethyl)amino)ethyl)-3,5-dimethyl-1H-pyrazol-4-yl)acetamido)ethoxy)-N2,N6-bis(4-(2-(dimethylamino)ethoxy)quinolin-2-yl)pyridine-2,6-dicarboxamide (PDF-Pz) (C_46_H_58_N_12_O_6_; MW = 875.03 gmol^−1^), and [Re(*k*^3^-pz-PDF)(CO)_3_]Br (PDF-Pz-Re) (C_49_H_58_N_12_O_9_ReBr; MW = 1225.17 gmol^−1^) were synthesized and characterized by HPLC, ESI-MS and multinuclear NMR as previously described [21]. Stock solutions of all compounds (20 mM) were prepared in DMSO. For biological assays, each stock solution was diluted with culture medium to achieve the desired final concentrations.

### 3.2. Cell Culture and Cell Viability Assay

PC3 human prostate cancer cell line (ECACC) was grown at 37 °C in a humidified atmosphere of 5% CO_2,_ in Roswell Park Memorial Institute (RPMI, Gibco, Grand Island, NY, USA) 1640 culture medium supplemented with 10% heat-inactivated fetal bovine serum (FBS) and 1% penicillin–streptomycin. The antiproliferative activity of compound RHPS4 in PC3 cells was assessed by the evaluation of its effects on cellular proliferation using the [1-(4,5-dimethylthiazol-2-yl)-2,5-diphenyl tetrazolium] (MTT) assay, as previously described for PDS, PDF, PDF-Pz, and PDF-Pz-Re [21]. PC3 cells were seeded in 96-well culture plates at a density of 2 × 10^4^ cells/well and left to adhere overnight at 37  °C. Cells were then incubated with the desired RHPS4 concentrations (0.5, 1, 2, 5, 10, 20, and 100 μM) for 48 h at 37  °C. After incubation, the cells were washed with PBS and then incubated with MTT (0.5 mg/mL in PBS) for 3 h at 37  °C. Following removal of the MTT solution, the resulting insoluble blue formazan crystals were dissolved in DMSO. Absorbance was measured at 570 nm using a plate spectrophotometer (Power Wave Xs; Bio-Tek, Winooski, VT, USA). Each experiment was conducted with a minimum of six replicates. Growth inhibition (%) was calculated relative to a control sample incubated without the test compound. IC_50_ values—defined as the concentration required to inhibit cell growth by 50%—were determined using GraphPad Prism 10 software and reported in micromolar (µM) concentrations.

### 3.3. Cellular Irradiations

The irradiation of PC3 cells was performed in a Precisa-22 (Graviner Manufacturing Company Ltd., Gosport, UK) ^60^Co irradiator installed at Campus Tecnológico e Nuclear/Instituto Superior Técnico (CTN/IST) [38]. The Co-60 chamber is composed of four Co-60 sources with a total activity of 59.459 TBq (January 2022). Precisa-22 is equipped with an automated sample rotation system that allows for higher homogeneity of dose distribution across samples, higher reproducibility of irradiation experiments and a broad range of dose rates. The dose rate, used for cellular irradiations, was approximately 1 Gy/min as assessed by ionization chamber (Farmer type chamber FC65-P) measurements.

### 3.4. Radiobiological Effects

#### 3.4.1. Cell Proliferation and Colony Formation Assay

PC3 cells were seeded in 6-well culture plates (50, 100, and 200 cells for control conditions, and 100, 200, and 400 cells per well for ^60^Co exposure) and allowed to attach for 24 h. PC3 cells were incubated with the desired concentration (see Table 1) of each G4-binder for 48 h, and then irradiated with 0.5, 1, 2, 4, and 6 Gy of γ-radiation (dose rate of 1 Gy/min). Similar IR-only experiments were performed using the same radiation doses, i.e., without previously incubating the cells with the compounds.

After irradiation, the PC3 cells’ proliferation was evaluated by the clonogenic assay, with addition of fresh medium to the cells that were then incubated for 14 days in the same conditions. After 14 days of incubation, the medium was aspirated and cells were fixed and stained with Giemsa’s azur eosin methylene blue solution diluted 5% *v*/*v* in buffer solution.

The data of the clonogenic assay were used to obtain experimental survival curves that were fitted using the LQ model [27], according to the following formula:

−lnS = ⍺D + βD^2^ (1)

where S is the surviving fraction of the cells, and α and β are the proportionality factors of the absorbed dose (D) (Gy^−1^) and the dose squared (D^2^) (Gy^−2^), respectively.

#### 3.4.2. ɣ-H2AX Assay and Foci Analysis

PC3 cells were seeded at a density of 10,000 cells per well in an eight-well chamber slide and allowed to adhere overnight. Cells were then treated with 30 μM of either PDF-Pz or PDF-Pz-Re for 48 h at 37 °C, followed by exposure to 2 or 4 Gy of γ-radiation. Post-irradiation, cells were washed three times with PBS and fixed with 4% formaldehyde in PBS for 15 min. Permeabilization was performed using 0.5% Triton X-100 at room temperature for 5 min, followed by two washes with 1% BSA in PBS. Next, cells were incubated with a primary anti-γ-H2AX antibody (mouse anti-γ-H2AX (Ser139), Stressgen, San Diego, CA, USA) at 2 μg/mL for 1 h. After two washes with 1% BSA in PBS, cells were incubated with a Texas Red-X-conjugated anti-mouse secondary antibody at 1 mg/mL for 1 h, followed by three PBS washes. Nuclei were counterstained using DAPI in anti-fade mounting medium (Vectashield H-1200, Vector Laboratories, Burlingame, CA, USA). Imaging was performed at 64× magnification. Several high-resolution 2D images were randomly acquired from each slide and analyzed using the Speckle Count pipeline in CellProfiler [39]. At least 200 nuclei were evaluated per experimental condition. Statistical analysis was conducted using GraphPad prism 10 software.

### 3.5. Production of ROS

Intracellular ROS levels in PC3 cells were assessed using the cell-permeant probe 2′,7′-dichlorofluorescein diacetate (H_2_DCF-DA). Once inside the cells, intracellular esterases cleave the acetate groups, converting H_2_DCF-DA into non-fluorescent H_2_DCF, which is subsequently oxidized by reactive oxygen species (ROS) to form the highly fluorescent compound 2′,7′-dichlorofluorescein (DCF) [40]. PC3 cells (2 × 10^4^ cells/well) were seeded into 96-well plates and allowed to adhere overnight. The following day, cells were treated with 30 μM of either PDF-Pz or PDF-Pz-Re for 48 h at 37 °C, followed by exposure to 2 or 4 Gy of γ-radiation. Immediately after irradiation, the medium was replaced with 10 μM H_2_DCF-DA diluted in FluoroBrite™ DMEM (Gibco, Grand Island, NY, USA), and cells were incubated for 30 min at 37 °C. The medium was then aspirated, and cells were washed with PBS. DCF fluorescence was measured using a Varioskan Lux multimode microplate reader (Thermo Fisher Scientific, Waltham, MA, USA) with excitation at 492 nm and emission at 517 nm. Hydrogen peroxide (H_2_O_2_, 10 µM, incubation time of 20 min) was used as a positive control. Fluorescence results were expressed as fold changes relative to untreated control samples.

## 4. Conclusions

The radiosensitizing effects of PDF-Pz and PDF-Pz-Re in PC3 cells were evaluated in comparison with other G4 ligands, including related pyridostatin derivatives (PDS and PDF) and RHPS4, which has been reported as a potent radiosensitizer in various cancer cell types. The combination of PDF-Pz and PDF-Pz-Re with Co-60 radiation enhanced PC3 cell killing and DNA damage, demonstrating that these compounds are capable of ra-diosensitizing this cell line, albeit to a lesser extent than RHPS4.

The EF(α) value, corresponding to the enhancement factor for the alpha component of the LQ model, found for PDF-Pz and PDF-Pz-Re in PC3 cells was 1.7 ± 0.6 and 1.4 ± 0.5, respectively. This factor is pivotal to quantifying the increase in cellular sensitivity to radiation, particularly related to complex DNA lesions, and the obtained values confirm the radiosensitizing capabilities of PDF-Pz-Re and its chelator. Most importantly, PDF-Pz-Re offers the possibility to explore theranostic approaches in combination with its ^99m^Tc congener (PDF-Pz-^99m^Tc), which is suitable for in vivo SPECT imaging. These favorable features encourage further preclinical studies with these organometallic complexes, including SPECT imaging (^99m^Tc) and radiosensitization assays (Re) in tumor animal models.

All in all, our results corroborate that G4 DNA ligands represent a promising class of radiosensitizers, deserving further studies to optimize their therapeutic potential and to elucidate their mechanisms of action in cancer cells.

## Figures and Tables

**Table 1:** IC50 values and concentration of each G4-binder used in the irradiation experiments.

G4-Binder	IC50 (µM)	[G4-Binder] (µM) Used in the Irradiation Experiments
PDS	43.8 ± 25.5	20
PDF	10.7 ± 2.2	5
PDF-Pz	64.6 ± 24.9	30
PDF-Pz-Re	41.6 ± 8.8	30
RHPS4	8.7 ± 1.9	2.5

**Table 2:** Values of the LQ parameters α and β, radiation dose at 10% survival (D_10_) and enhancement factor (EF) for PC3 cells treated only with Co-60 irradiation and submitted to combined treatment with IR and with the different G4-binders under study. The EF was calculated both for 10% of SF and for ⍺ and β parameters. The values represent the mean ± SD of three independent experiments.

	Control	PDS	PDF	PDF-Pz	PDF-Pz-Re	RHPS4
α [Gy^−1^]	0.197 ± 0.061	0.271 ± 0.100	0.275 ± 0.025	0.325 ± 0.045	0.270 ± 0.063	0.375 ± 0.035
β [Gy^−2^]	0.007 ± 0.015	0.003 ± 0.026	0.004 ± 0.015	0.005 ± 0.015	0.003 ± 0.016	0.001 ± 0.010
D_10_ (Gy)	8.9 ± 3.0	7.8 ± 4.6	7.5 ± 1.7	6.4 ± 1.5	7.8 ± 2.5	6.1 ± 1.1
EF (D_10_)	-	1.1 ± 0.8	1.2 ± 0.5	1.4 ± 0.6	1.1 ± 0.5	1.5 ± 0.6
EF (⍺)	-	1.4 ± 0.7	1.4 ± 0.5	1.7 ± 0.6	1.4 ± 0.5	1.9 ± 0.6
EF (β)	-	0.4 ± 3.8	0.5 ± 2.4	0.7 ± 2.6	0.4 ± 2.4	0.1 ± 1.5

## Data Availability

The raw data presented in this study are available on request from the corresponding author.

## Abbreviations

The following abbreviations are used in this manuscript:

D_10_	Radiation dose at 10% survival
DAPI	4′,6-diamidino-2-phenylindole,
DCF	2′,7′-dichlorofluorescein
DDR	DNA damage response
DMSO	Dimethyl sulfoxide
DSBs	Double-strand breaks
EBRT	External beam radiation therapy
EF	Enhancement factor
G4	G-quadruplex
H2DCF-DA	2′,7′-dichlorodihydrofluorescein diacetate
HPLC	High Performance Liquid Chromatography
IC50	Half maximal inhibitory concentration
IR	Ionizing radiation
LQ	Linear-quadratic
MTT	3-(4,5-dimethylthiazol-2-yl)-2,5-diphenyltetrazolium bromide
PBS	Phosphate buffered saline
NMR	Nuclear magnetic resonance
PCa	Prostate cancer
PDF	4-(2-aminoethoxy)-N2,N6-bis(4-(2-(dimethylamino)ethoxy)quinolin-2-yl)pyridine-2,6-dicarboxamide
PDF-Pz	4-(2-(2-(1-(2-((2-aminoethyl)amino)ethyl)-3,5-dimethyl-1H-pyrazol-4-yl)acetamido)ethoxy)-N2,N6-bis(4-(2-(dimethylamino)ethoxy)quinolin-2-yl)pyridine-2,6-dicarboxamide
PDS	4-(2-aminoethoxy)-N2,N6-bis [4-(2-aminoethoxy)-2-quinolinyl]-2,6-pyridinedicarboxamide hydrochloride
ROS	Reactive oxygen species
SF	Survival fraction
